# Penicillin- and Ciprofloxacin-Resistant Invasive Neisseria meningitidis Isolates from Japan

**DOI:** 10.1128/spectrum.00627-22

**Published:** 2022-04-25

**Authors:** Ryoichi Saito, Jun Nakajima, Isaac Prah, Masatomo Morita, Samiratu Mahazu, Yusuke Ota, Ayuka Kobayashi, Shuji Tohda, Hajime Kamiya, Hideyuki Takahashi, Makoto Ohnishi

**Affiliations:** a Department of Molecular Microbiology, Graduate School of Medicine and Dental Science, Tokyo Medical and Dental University, Tokyo, Japan; b Department of Clinical Laboratory, Tokyo Medical and Dental University Hospital, Tokyo, Japan; c Department of Bacteriology I, National Institute of Infectious Diseases, Tokyo, Japan; d Infectious Disease Surveillance Center, National Institute of Infectious Diseases, Tokyo, Japan; e National Institute of Infectious Diseases, Tokyo, Japan; University of Arizona/Banner Health

**Keywords:** *Neisseria meningitidis*, chemoprophylaxis, ciprofloxacin resistance, invasive lineage, invasive meningococcal disease, penicillin resistance

## Abstract

Neisseria meningitidis causes a life-threatening invasive meningococcal disease (IMD). Isolates resistant to antibiotics, such as penicillin, ceftriaxone, and ciprofloxacin that are recommended for the treatment of IMD patients and their close contacts have been serious public health concerns globally. However, susceptibility profiles to critically important antibiotics and the genetic characteristics of isolates possessing antibiotic resistance are extremely limited as IMD incidence is low in Japan. We assessed the susceptibility profiles of 87 randomly selected, sterile site-derived N. meningitidis strains isolated from hospitals nationwide, recovered between April 1998 and March 2018 in Japan, to seven antibiotics. As a result, we demonstrated, for the first time, that the isolates remained highly susceptible to ceftriaxone, meropenem, azithromycin, ciprofloxacin, chloramphenicol, and rifampin, but not to penicillin. We then characterized the genetic relatedness of six penicillin- and/or ciprofloxacin-resistant isolates obtained in this study with global 112 genomes using core-genome phylogenetic analysis. These results provide the first evidence that invasive lineages such as a penicillin-resistant serogroup W, sequence type (ST)-11 clonal complex (CC), and a ciprofloxacin-resistant serogroup B/C, ST-4821 CC that is considered as a global threat, have been sporadically identified in Japan. Our findings highlight the need to monitor antibiotic resistance in clinical isolates of N. meningitidis, thereby preventing the spread of antibiotic-resistant invasive lineages and maintaining effective treatment for IMD patients and their close contacts.

**IMPORTANCE** Although antibiotics such as penicillin and ceftriaxone can treat invasive meningococcal disease (IMD), the emergence and spread of antibiotic-resistant Neisseria meningitidis have become a global concern. To provide effective treatment, including chemoprophylaxis to IMD patients and their close contacts, we highlighted the importance of recognizing the antibiotic resistance and genetic features of N. meningitidis isolates.

## OBSERVATION

Invasive meningococcal disease (IMD) is a well-known life-threatening illness caused by the bacterium Neisseria meningitidis. N. meningitidis strains with resistance to antibiotics, such as penicillin, ciprofloxacin, and ceftriaxone, that are recommended for the treatment and chemoprophylaxis of IMD patients and their close contacts, are reported globally and raise serious public health concerns worldwide owing to limited options for treatment (1–9). However, data such as the resistance rate to critically important antibiotics and the genetic relationship between isolates collected are extremely scarce in Japan, as IMD incidence is low (0.028 cases/100,000 persons per year in 2014) (10). This situation may cause inadequate treatment of IMD patients and make management of antibiotic-resistant strains more difficult based on genetic relatedness in Japan when those strains are disseminated. Here, we described the antibiotic susceptibility profiles of 87 N. meningitidis isolates recovered during a national surveillance conducted for approximately 20 years in Japan and provided insights on the genetic features of penicillin- and/or ciprofloxacin-resistant isolates.

A total of 87 nonduplicate N. meningitidis isolates (blood, *n* = 62; cerebrospinal fluid, *n* = 22; synovial fluid, *n* = 2; aqueous humor, *n* = 1) were randomly selected from over 200 sterile site-derived isolates obtained between April 1998 and March 2018 at the National Institute of Infectious Diseases, Japan (Table S1 in the supplemental material). Microbial identification was performed using biochemical profiling (ID-test HN-20 Rapid System; Nissui Pharmaceutical, Tokyo, Japan) and the MALDI Biotyper (Bruker Daltonics, Karlsruhe, Germany). Serogroup determination and multilocus sequence typing (MLST) were performed as previously described (11). The MICs of penicillin, ceftriaxone, meropenem, azithromycin, ciprofloxacin, chloramphenicol, and rifampin were determined using an Etest (bioMérieux, Marcy-l’Étoile, France) on Mueller-Hinton agar with 5% sheep blood (Becton, Dickinson and Company, Franklin Lakes, NJ) according to the manufacturer’s instructions. Quality control was performed using Streptococcus pneumoniae ATCC 49619 and Escherichia coli ATCC 25922. Results were interpreted according to the Clinical and Laboratory Standards Institute guideline M100-ED30 (12). β-lactamase production among strains that were nonsusceptible to penicillin was determined using BBL Cefinase paper discs (Becton, Dickinson and Company).

Among the 87 isolates, 41 (47.1%) were nonsusceptible to penicillin, including four resistant isolates (4.6%), eight (9.2%) were nonsusceptible to ciprofloxacin, including five resistant isolates (5.7%), and three (3.4%) were nonsusceptible to azithromycin (Table 1). Isolates NIID576, NIID614, and NIID620 were resistant to both penicillin and ciprofloxacin (Table S1). β-lactamase production was negative in all penicillin-nonsusceptible isolates. The geometric mean MICs tended to increase for penicillin and ciprofloxacin every seven years, due to the increasing rate of antibiotic-intermediate and -resistant isolates between 2012 and 2018 (Fig. S1), whereas the geometric mean MIC for azithromycin tended to decrease. No isolate was found nonsusceptible or resistant to ceftriaxone, meropenem, chloramphenicol, or rifampin. These results agree with previous studies that showed reduced susceptibility to penicillin among invasive isolates (7–9, 13, 14), suggesting that penicillin might not be suitable to treat IMD cases in Japan. However, we could not clarify the reason for the observation in this study, and it might have been associated with the antibiotic choice for the treatment of bacterial meningitis in Japan. Furthermore, we provide the first evidence that clinical isolates from Japan for approximately 20 years remain highly susceptible to critically important antibiotics, except for penicillin, that are currently used for treatment and chemoprophylaxis of IMD patients and their close contacts.

**TABLE 1:** Antibiotic susceptibility profiles of the 87 N. meningitidis isolates used in this study

Antibiotic	No.	MIC (mg/L)			Interpretative category^*a*^		
		Range	MIC_50_	MIC_90_	%S	%I	%R (% non-S)
Penicillin	87	0.031–0.5	0.063	0.125	52.9	42.5	4.6
Ceftriaxone	87	≤0.004	≤0.004	≤0.004	100.0	(0.0)	(0.0)
Meropenem	87	≤0.004–0.063	0.008	0.016	100.0	(0.0)	(0.0)
Azithromycin	87	0.125–4	1	2	96.6	(0.0)	(3.4)
Ciprofloxacin	87	≤0.004–0.25	≤0.004	0.008	90.8	3.4	5.7
Chloramphenicol	87	0.5–2	1	2	100.0	0.0	0.0
Rifampicin	87	≤0.004–0.25	0.008	0.063	100.0	0.0	0.0

a S, susceptible; I, intermediate; R, resistant. The category was interpreted by the Clinical and Laboratory Standards Institute guideline M100-ED30.

Next, to assess the genetic features of our six penicillin- and/or ciprofloxacin-resistant isolates defined using the CLSI guideline (12), we conducted whole-genome sequencing. Genomic DNA from the six isolates was extracted using the NucleoSpin tissue kit (TaKaRa Bio, Shiga, Japan). The DNA library was prepared using the Nextera XT DNA library preparation kit and sequenced on MiSeq (Illumina, San Diego, CA) generating a 2 × 300-bp paired-end read. The genome coverages were 77.1× to 186.1× among the six isolates. Genome assembly was performed using the SPAdes, v3.13.1 with default parameters (15). Subsequently, gene annotation was performed using Prokka v1.11 (16), and the sequence data were submitted to the PubMLST *Neisseria* genome database (17) and the DDBJ Sequence Read Archive. Moreover, to determine genetic relatedness among the 6 genomes obtained in this study and 112 genomes from global N. meningitidis strains categorized as penicillin- and ciprofloxacin-resistant (MICs ≥ 0.5 and ≥ 0.12 mg/L, respectively) (12) deposited in the PubMLST *Neisseria* genome database (Table S2; assessed June 11, 2021), a core-genome based phylogeny with 1,000 bootstrap replicates was constructed using IQ-TREE v1.6.12 (18). The tree was visualized and annotated using iTOL v6 (19). Most of the strains were isolated from usually sterile sites between 1999 and 2020.

**FIG 1:**
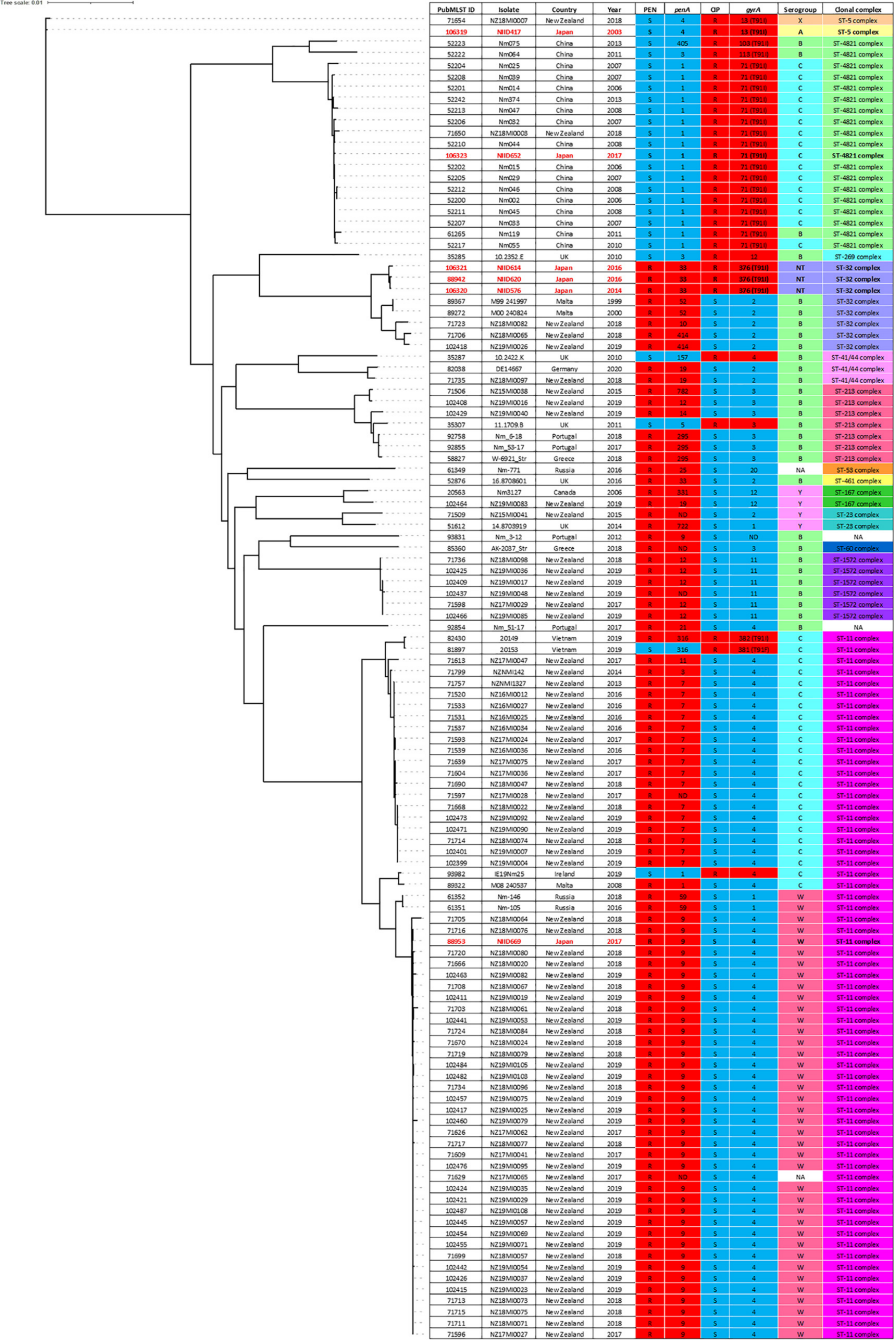
A core-genome based phylogeny and data of six (labeled in red from this study) and 112 (global collection) penicillin- and/or ciprofloxacin-resistant N. meningitidis genomes. The maximum-likelihood phylogenetic tree with 1,000 bootstrap replicates was generated. Data on penicillin and ciprofloxacin categories, serogroup, and clonal complex are shown with the year and place of isolation and the *penA* and *gyrA* alleles. A T91I substitution responsible for ciprofloxacin resistance is also included alongside *gyrA.* ND, not determined; NA, not available.

A penicillin-resistant isolate NIID669 was classified into serogroup W, ST-11 clonal complex (CC) harboring *penA9*, a mosaic *penA* allele that has been reported in strains with reduced penicillin susceptibility (6, 9, 14), which clustered to a clade of the same serogroup and CC containing penicillin-resistant strains from New Zealand (*penA9*) (Fig. 1). This indicates, for the first time, that the penicillin-resistant invasive serogroup W, ST-11 CC lineage circulating in several countries (5, 6) has already emerged in Japan. Three penicillin and ciprofloxacin-resistant isolates, NIID576, NIID614, and NIID620, recovered from individuals in different prefectures, were classified as the same nontypeable serogroup ST-32 CC strains harboring both *penA33* (identified previously in penicillin-nonsusceptible strains) (14) and *gyrA376* with a T91I substitution responsible for ciprofloxacin resistance (Fig. 1). These isolates were clustered with the penicillin-resistant and ciprofloxacin-susceptible serogroup B, ST-32 CC strains identified in Malta and New Zealand. This highlights the necessity to monitor this lineage containing nontypeable serogroup strains due to the possible difficulty in preventing disease with vaccines available worldwide. A ciprofloxacin-resistant isolate NIID417 classified into serogroup A, ST-5 CC, harboring *gyrA13* with a T91I substitution, had a close phylogenetic distance with a strain from New Zealand that also shared the same ciprofloxacin resistance mechanism but had a different serogroup identity (Fig. 1). The remaining ciprofloxacin-resistant isolate NIID652 was classified into serogroup C, ST-4821 CC, harboring *gyrA71* with a T91I substitution, and clustered to a clade composed of serogroup B/C and the same CC with ciprofloxacin resistance, obtained from China and New Zealand (Fig. 1). Invasive strains with ciprofloxacin resistance, which belong to ST-4821 CC, ST-23 CC, and ST-175 CC with alleles mostly containing a T91I substitution, have been observed in several countries, including Japan (1–4). Although our five ciprofloxacin-resistant isolates, including the ST-32 CC strains, could have sporadically emerged, they are not widespread in Japan as of now; however, considering the current global concerns, these findings emphasize the need for a continuous and wider surveillance to prevent the circulation of ciprofloxacin-resistant clones in Japan.

Azithromycin is thought of as an alternative chemoprophylaxis in case of ciprofloxacin resistance (20). As three isolates nonsusceptible to azithromycin have already sporadically emerged in Japan, as shown in this study, the monitoring of resistance to azithromycin will be required for maintaining the effective chemoprophylaxis strategy for close contact individuals of IMD patients.

A major limitation of our study is that our results may not reflect the entire IMD scenario in Japan during the study period since the collection of strains from meningococcal meningitis cases is still not regulated by the Infectious Diseases Control Law in Japan although the patients’ records of meningococcal meningitis were collected as a notifiable disease as per the law from 1999. We investigated the phylogenetic relationship of our penicillin- and ciprofloxacin-resistant isolates with international strains derived only from the PubMLST database, and these data sets extracted may be prone to biases for regions. Analysis with a higher number of global strains would have helped to better appreciate the global distribution and genetic relatedness among them. Moreover, this study focused on the phenotype and genotype of N. meningitidis isolates in Japan but not the mechanisms underlying antibiotic resistance.

In conclusion, we discovered that N. meningitidis isolates recovered nationwide in Japan remained highly susceptible to antibiotics typically recommended for IMD patients and their close contacts, except for penicillin. However, invasive lineages with penicillin and/or ciprofloxacin resistance considered as public threats in several countries have been sporadically identified in Japan. Therefore, our findings highlight the importance of monitoring antibiotic resistance in preventing its spread, maintaining effective treatment and management of IMD patients and their close contacts, and improving infection control.

### Data availability.

The genomes have been deposited in GenBank under the BioSample accession numbers SAMD00391203 (NIID417), SAMD00320028 (NIID576), SAMD00320050 (NIID614), SAMD00320056 (NIID620), SAMD00286455 (NIID652), and SAMD00286469 (NIID669).

## References

[1] Willerton L, Lucidarme J, Campbell H, Caugant DA, Claus H, Jacobsson S, Ladhani SN, Molling P, Neri A, Stefanelli P, Taha MK, Vogel U, Borrow R. 2020. Geographically widespread invasive meningococcal disease caused by a ciprofloxacin resistant non-groupable strain of the ST-175 clonal complex. J Infect 81:575–584. doi:10.1016/j.jinf.2020.08.030. 32858070

[2] Kawasaki Y, Matsubara K, Takahashi H, Morita M, Ohnishi M, Hori M, Isome K, Iwata A, Nigami H, Ikemachi M, Yamamoto G, Ohkusu K. 2018. Invasive meningococcal disease due to ciprofloxacin-resistant *Neisseria meningitidis* sequence type 4821: the first case in Japan. J Infect Chemother 24:305–308. doi:10.1016/j.jiac.2017.11.001. 29233459

[3] Zhu B, Fan Y, Xu Z, Xu L, Du P, Gao Y, Shao Z. 2014. Genetic diversity and clonal characteristics of ciprofloxacin-resistant meningococcal strains in China. J Med Microbiol 63:1411–1418. doi:10.1099/jmm.0.078600-0. 25082942

[4] Tsang RS, Law DK, Deng S, Hoang L. 2017. Ciprofloxacin-resistant *Neisseria meningitidis* in Canada: likely imported strains. Can J Microbiol 63:265–268. doi:10.1139/cjm-2016-0716. 28140652

[5] Mowlaboccus S, Jolley KA, Bray JE, Pang S, Lee YT, Bew JD, Speers DJ, Keil AD, Coombs GW, Kahler CM. 2017. Clonal expansion of new penicillin-resistant clade of *Neisseria meningitidis* serogroup W clonal complex 11, Australia. Emerg Infect Dis 23:1364–1367. doi:10.3201/eid2308.170259. 28609259 PMC5547816

[6] Willerton L, Lucidarme J, Walker A, Lekshmi A, Clark SA, Gray SJ, Borrow R. 2021. Increase in penicillin-resistant invasive meningococcal serogroup W ST-11 complex isolates in England. Vaccine 39:2719–2729. doi:10.1016/j.vaccine.2021.03.002. 33858720

[7] Hedberg ST, Fredlund H, Nicolas P, Caugant DA, Olcen P, Unemo M. 2009. Antibiotic susceptibility and characteristics of *Neisseria meningitidis* isolates from the African meningitis belt, 2000 to 2006: phenotypic and genotypic perspectives. Antimicrob Agents Chemother 53:1561–1566. doi:10.1128/AAC.00994-08. 19188396 PMC2663058

[8] Harcourt BH, Anderson RD, Wu HM, Cohn AC, MacNeil JR, Taylor TH, Wang X, Clark TA, Messonnier NE, Mayer LW. 2015. Population-based surveillance of *Neisseria meningitidis* antimicrobial resistance in the United States. Open Forum Infect Dis 2:ofv117. doi:10.1093/ofid/ofv117. 26357666 PMC4561371

[9] Bertrand S, Carion F, Wintjens R, Mathys V, Vanhoof R. 2012. Evolutionary changes in antimicrobial resistance of invasive *Neisseria meningitidis* isolates in Belgium from 2000 to 2010: increasing prevalence of penicillin nonsusceptibility. Antimicrob Agents Chemother 56:2268–2272. doi:10.1128/AAC.06310-11. 22290951 PMC3346653

[10] Aye AMM, Bai X, Borrow R, Bory S, Carlos J, Caugant DA, Chiou CS, Dai VTT, Dinleyici EC, Ghimire P, Handryastuti S, Heo JY, Jennison A, Kamiya H, Tonnii Sia L, Lucidarme J, Marshall H, Putri ND, Saha S, Shao Z, Sim JHC, Smith V, Taha MK, Van Thanh P, Thisyakorn U, Tshering K, Vazquez J, Veeraraghavan B, Yezli S, Zhu B. 2020. Meningococcal disease surveillance in the Asia-Pacific region (2020): the global meningococcal initiative. J Infect 81:698–711. doi:10.1016/j.jinf.2020.07.025. 32730999

[11] Takahashi H, Kuroki T, Watanabe Y, Tanaka H, Inouye H, Yamai S, Watanabe H. 2004. Characterization of *Neisseria meningitidis* isolates collected from 1974 to 2003 in Japan by multilocus sequence typing. J Med Microbiol 53:657–662. doi:10.1099/jmm.0.45541-0. 15184538

[12] Clinical and Laboratory Standards Institute. 2020. Performance standards for antimicrobial susceptibility testing, 30th ed. CLSI, Wayne, PA, USA.

[13] Gorla MC, Pinhata JMW, Dias UJ, de Moraes C, Lemos AP. 2018. Surveillance of antimicrobial resistance in *Neisseria meningitidis* strains isolated from invasive cases in Brazil from 2009 to 2016. J Med Microbiol 67:750–756. doi:10.1099/jmm.0.000743. 29717974

[14] Willerton L, Lucidarme J, Walker A, Lekshmi A, Clark SA, Walsh L, Bai X, Lee-Jones L, Borrow R. 2021. Antibiotic resistance among invasive *Neisseria meningitidis* isolates in England, Wales and Northern Ireland (2010/11 to 2018/19). PLoS One 16:e0260677. doi:10.1371/journal.pone.0260677. 34843604 PMC8629238

[15] Bankevich A, Nurk S, Antipov D, Gurevich AA, Dvorkin M, Kulikov AS, Lesin VM, Nikolenko SI, Pham S, Prjibelski AD, Pyshkin AV, Sirotkin AV, Vyahhi N, Tesler G, Alekseyev MA, Pevzner PA. 2012. SPAdes: a new genome assembly algorithm and its applications to single-cell sequencing. J Comput Biol 19:455–477. doi:10.1089/cmb.2012.0021. 22506599 PMC3342519

[16] Seemann T. 2014. Prokka: rapid prokaryotic genome annotation. Bioinformatics 30:2068–2069. doi:10.1093/bioinformatics/btu153. 24642063

[17] Jolley KA, Bray JE, Maiden MCJ. 2018. Open-access bacterial population genomics: BIGSdb software, the PubMLST.org website and their applications. Wellcome Open Res 3:124. doi:10.12688/wellcomeopenres.14826.1. 30345391 PMC6192448

[18] Prah I, Ayibieke A, Mahazu S, Sassa CT, Hayashi T, Yamaoka S, Suzuki T, Iwanaga S, Ablordey A, Saito R. 2021. Emergence of oxacillinase-181 carbapenemase-producing diarrheagenic *Escherichia coli* in Ghana. Emerg Microbes Infect 10:865–873. doi:10.1080/22221751.2021.1920342. 33879019 PMC8110189

[19] Letunic I, Bork P. 2019. Interactive Tree of Life (iTOL) v4: recent updates and new developments. Nucleic Acids Res 47:W256–W259. doi:10.1093/nar/gkz239. 30931475 PMC6602468

[20] Krone M, Lam TT, Vogel U, Claus H. 2020. Susceptibility of invasive *Neisseria meningitidis* strains isolated in Germany to azithromycin, an alternative agent for post-exposure prophylaxis. J Antimicrob Chemother 75:984–987. doi:10.1093/jac/dkz535. 31971241

