# Supplementary material for: Penicillin- and Ciprofloxacin-Resistant Invasive Neisseria meningitidis Isolates from Japan
Source: Microbiol Spectr. 2022 Apr 25;10(3):e00627-22. doi: 10.1128/spectrum.00627-22 (PMC9241714; doi:10.1128/spectrum.00627-22)

TABLE S1 Antibiotic susceptibility, serogroup, and sequence type of 87 *N. meningitidis* isolates used in this study.

Strain	Year	Source	MIC (mg/L)							Serogroup	Sequence type
			Penicillin	Ceftriaxone	Meropenem	Azithromycin	Ciprofloxacin	Chloramphenicol	Rifampicin		
NIID81	1998	Cerebrospinal fluid	0.125	≤0.004	0.006	1.00	≤0.004	0.75	0.004	Y	ND
NIID90	2000	Cerebrospinal fluid	0.125	≤0.004	0.006	0.75	≤0.004	0.75	0.012	Y	ND
NIID287	1998	Blood	0.047	≤0.004	0.004	0.75	≤0.004	0.75	0.004	B	437
NIID343	2000	Blood	0.094	≤0.004	0.008	0.75	≤0.004	0.75	0.012	Y	23
NIID345	2000	Cerebrospinal fluid	0.064	≤0.004	0.004	0.750	≤0.004	0.750	0.006	B	687
NIID375	2001	Cerebrospinal fluid	0.047	≤0.004	0.006	1.50	≤0.004	1.00	0.023	B	1418
NIID392	2002	Blood	0.047	≤0.004	0.008	0.750	≤0.004	1.000	0.004	Y	2058
NIID393	2002	Blood	0.047	≤0.004	0.006	0.75	≤0.004	0.750	0.004	B	269
NIID398	2002	Blood	0.047	≤0.004	0.006	1.00	≤0.004	0.750	0.012	NT	803
NIID411	2003	Blood	0.047	≤0.004	0.004	1.000	≤0.004	1.000	0.008	B	1418
NIID412	2003	Cerebrospinal fluid	0.125	≤0.004	0.006	1.00	≤0.004	1.500	0.006	Y	23
NIID416	2003	Blood	0.190	≤0.004	0.008	1.50	≤0.004	1.500	0.008	Y	23
NIID417	2003	Cerebrospinal fluid	0.125	≤0.004	0.006	4.000	0.125	2.000	0.250	A	7
NIID418	2003	Cerebrospinal fluid	0.064	≤0.004	0.006	1.500	≤0.004	1.500	0.012	B	618
NIID419	2003	Blood	0.064	≤0.004	0.006	0.750	≤0.004	1.000	0.008	B	687
NIID422	2003	Blood	0.094	≤0.004	0.006	2.00	≤0.004	1.000	0.032	B	3015
NIID425	2004	Cerebrospinal fluid	0.023	≤0.004	0.004	0.500	≤0.004	0.380	0.023	B	687
NIID427	2004	Cerebrospinal fluid	0.064	≤0.004	0.004	1.50	≤0.004	1.000	0.012	B	3506
NIID429	2004	Blood	0.094	≤0.004	0.006	2.00	≤0.004	2.000	0.023	Y	23
NIID430	2004	Cerebrospinal fluid	0.047	≤0.004	0.004	1.000	≤0.004	0.750	0.016	B	687
NIID434	2004	Cerebrospinal fluid	0.064	≤0.004	0.004	1.000	≤0.004	1.000	0.008	B	2162
NIID458	2006	Cerebrospinal fluid	0.064	≤0.004	0.006	1.500	≤0.004	1.500	0.047	Y	5168
NIID461	2006	Cerebrospinal fluid	0.047	≤0.004	0.004	0.750	≤0.004	1.000	0.008	B	3495
NIID464	2006	Blood	0.094	≤0.004	0.006	1.500	≤0.004	1.500	0.064	Y	3015
NIID465	2006	Blood	0.064	≤0.004	0.008	0.750	≤0.004	0.750	0.032	Y	23
NIID466	2006	Cerebrospinal fluid	0.125	≤0.004	0.008	1.500	≤0.004	1.500	0.012	Y	23
NIID472	2006	Cerebrospinal fluid	0.125	≤0.004	0.008	1.500	≤0.004	1.500	0.012	Y	23
NIID485	2007	Blood	0.047	≤0.004	0.004	1.000	≤0.004	1.000	0.016	B	32
NIID496	2009	Blood	0.094	≤0.004	0.012	0.500	≤0.004	1.500	0.016	Y	23
NIID500	2009	Blood	0.094	≤0.004	0.008	1.000	≤0.004	1.500	0.032	NT	23
NIID501	2009	Cerebrospinal fluid	0.064	≤0.004	0.008	1.000	≤0.004	1.000	0.004	Y	1655
NIID502	2009	Blood	0.047	≤0.004	0.006	0.380	≤0.004	0.750	0.016	Y	23
NIID503	2009	Blood	0.032	≤0.004	0.003	1.000	≤0.004	1.000	0.004	B	4893
NIID518	2011	Blood	0.064	≤0.004	0.008	0.750	≤0.004	1.000	0.012	Y	23
NIID530	2011	Blood	0.064	≤0.004	0.008	0.750	≤0.004	1.500	0.023	Y	23
NIID534	2011	Cerebrospinal fluid	0.064	≤0.004	0.006	2.000	≤0.004	1.500	0.047	Y	3015
NIID535	2011	Blood	0.190	≤0.004	0.006	1.50	0.008	1.0	0.008	W-135	11
NIID536	2012	Blood	0.190	≤0.004	0.012	1.00	≤0.004	0.75	0.006	W-135	184
NIID546	2013	Blood	0.094	≤0.004	0.008	0.50	≤0.004	0.5	0.004	Y	23
NIID551	2013	Blood	0.094	≤0.004	0.008	0.75	≤0.004	0.5	0.004	Y	1655
NIID552	2013	Blood	0.125	≤0.004	0.012	0.750	≤0.004	1.000	0.008	Y	23
NIID554	2013	Blood	0.064	≤0.004	0.008	0.750	≤0.004	1.500	0.064	Y	23
NIID555	2013	Cerebrospinal fluid	0.094	≤0.004	0.008	1.000	≤0.004	1.500	0.023	Y	23
NIID559	2013	Blood	0.047	≤0.004	0.006	1.000	≤0.004	1.000	0.008	Y	1655
NIID560	2014	Blood	0.023	≤0.004	0.008	0.094	≤0.004	0.750	0.012	C	11
NIID561	2014	Blood	0.094	≤0.004	0.008	1.000	≤0.004	1.500	0.012	Y	1655
NIID563	2014	Cerebrospinal fluid	0.064	≤0.004	0.008	1.000	≤0.004	1.500	0.008	Y	1655
NIID564	2014	Blood	0.064	≤0.004	0.008	0.750	≤0.004	1.500	0.008	Y	1655
NIID566	2014	Blood	0.125	≤0.004	0.006	1.000	≤0.004	1.500	0.006	Y	23
NIID567	2014	Blood	0.064	≤0.004	0.006	1.000	≤0.004	1.500	0.094	Y	3015
NIID569	2014	Blood	0.064	≤0.004	0.008	0.750	≤0.004	1.000	0.016	Y	1655
NIID570	2014	Blood	0.125	≤0.004	0.012	1.500	≤0.004	2.000	0.016	Y	23
NIID572	2014	Blood	0.047	≤0.004	0.006	2.000	≤0.004	1.000	0.008	B	687
NIID573	2014	Blood	0.064	≤0.004	0.008	0.750	≤0.004	1.500	0.023	Y	23
NIID576	2014	Synovial fluid	0.500	≤0.004	0.047	6.000	0.125	2.000	0.094	NT	11026
NIID577	2015	Blood	0.094	≤0.004	0.008	0.750	≤0.004	1.000	0.006	Y	1655
NIID578	2015	Blood	0.047	≤0.004	0.006	1.000	≤0.004	1.000	0.064	NT	198
NIID581	2015	Aqueous humor	0.094	≤0.004	0.012	1.000	≤0.004	1.000	0.008	Y	1655
NIID592	2015	Blood	0.064	≤0.004	0.008	0.750	≤0.004	1.000	0.004	Y	1655
NIID595	2015	Blood	0.064	≤0.004	0.008	0.500	≤0.004	1.000	0.008	NT	11448
NIID597	2015	Blood	0.094	≤0.004	0.006	1.000	≤0.004	1.500	0.012	Y	23
NIID599	2015	Synovial fluid	0.047	≤0.004	0.008	0.750	≤0.004	0.750	0.004	W-135	11
NIID608	2015	Blood	0.094	≤0.004	0.008	0.750	≤0.004	1.000	0.008	Y	23
NIID610	2016	Blood	0.064	≤0.004	0.006	1.000	≤0.004	1.000	0.006	Y	1655
NIID611	2016	Blood	0.064	≤0.004	0.008	1.000	≤0.004	1.000	0.016	Y	23
NIID613	2016	Cerebrospinal fluid	0.094	≤0.004	0.008	1.000	≤0.004	1.500	0.008	Y	23
NIID614	2016	Blood	0.500	≤0.004	0.032	2.000	0.125	1.000	0.032	NT	11026
NIID618	2016	Blood	0.094	≤0.004	0.008	1.000	≤0.004	1.000	0.006	Y	1655
NIID619	2016	Cerebrospinal fluid	0.064	≤0.004	0.006	1.000	0.064	1.000	0.023	Y	23
NIID620	2016	Blood	0.380	≤0.004	0.032	1.500	0.094	1.000	0.012	NT	11026
NIID621	2016	Blood	0.094	≤0.004	0.008	1.00	≤0.004	0.5	0.004	Y	1655
NIID623	2016	Blood	0.094	≤0.004	0.008	0.75	≤0.004	0.75	0.003	Y	1655
NIID624	2016	Blood	0.094	≤0.004	0.006	0.750	0.064	1.000	0.008	B	2057
NIID625	2016	Blood	0.064	≤0.004	0.006	1.000	≤0.004	1.500	0.006	B	1655
NIID628	2016	Blood	0.094	≤0.004	0.008	1.000	≤0.004	1.000	0.006	Y	1655
NIID630	2016	Blood	0.064	≤0.004	0.008	1.000	≤0.004	1.000	0.032	Y	1655
NIID633	2016	Cerebrospinal fluid	0.064	≤0.004	0.004	1.000	≤0.004	1.000	0.008	B	687
NIID641	2016	Blood	0.064	≤0.004	0.012	1.500	≤0.004	0.750	0.006	Y	1655
NIID652	2017	Blood	0.094	≤0.004	0.006	2.000	0.250	1.000	0.094	C	4821
NIID657	2017	Blood	0.064	≤0.004	0.006	1.500	≤0.004	1.500	0.006	B	687
NIID659	2017	Blood	0.094	≤0.004	0.008	1.000	≤0.004	1.000	0.006	Y	1655
NIID669	2017	Blood	0.380	≤0.004	0.032	4.000	≤0.004	1.500	0.047	W-135	11
NIID672	2017	Blood	0.023	≤0.004	0.006	1.00	≤0.004	0.5	0.19	Y	23
NIID684	2017	Blood	0.094	≤0.004	0.012	0.75	≤0.004	1.0	0.008	Y	23
NIID686	2017	Blood	0.094	≤0.004	0.012	1.00	≤0.004	0.75	0.008	Y	1655
NIID699	2018	Blood	0.094	≤0.004	0.006	0.750	0.064	1.000	0.023	B	2057
NIID700	2018	Blood	0.064	≤0.004	0.006	1.500	≤0.004	1.000	0.006	Y	1655

NT, non-typable; ND, not determined.

TABLE S2 Data for 6 (this study) and 112 (global collection) penicillin- and/or ciprofloxacin-resistant *N. meningitidis* genomes.

PubMLST ID	Isolate	Contigs	Total length
71654	NZ18MI0007	216	2116570
106319	NIID417	86	2,136,740
52223	Nm075	156	2159040
52222	Nm064	121	2166275
52204	Nm025	108	2146819
52208	Nm039	118	2145513
52201	Nm014	105	2207007
52242	Nm374	106	2163637
52213	Nm047	107	2082666
52206	Nm032	292	2208937
71650	NZ18MI0003	195	2091996
52210	Nm044	93	2147190
106323	NIID652	84	2,097,035
52202	Nm015	106	2216362
52205	Nm029	102	2229869
52212	Nm046	116	2115259
52200	Nm002	103	2258893
52211	Nm045	122	2138642
52207	Nm033	109	2162440
61265	Nm119	1	2223033
52217	Nm055	121	2148520
35285	10.2352.E	193	2173911
106321	NIID614	96	2,223,543
88942	NIID620	90	2,180,008
106320	NIID576	77	2,189,331
89367	M99 241997	128	2195190
89272	M00 240824	142	2194311
71723	NZ18MI0082	209	2162717
71706	NZ18MI0065	166	2129476
102418	NZ19MI0026	238	2132787
35287	10.2422.K	182	2210189
82038	DE14667	278	2097481
71735	NZ18MI0097	167	2095491
71506	NZ15MI0038	285	2194577
102408	NZ19MI0016	280	2155159
102429	NZ19MI0040	279	2161774
35307	11.1709.B	225	2204802
92758	Nm_6-18	289	2213134
92855	Nm_53-17	246	2194138
58827	W-6921_Str	166	2071362
61349	Nm-771	80	2079516
52876	16.8708601	190	2255387
20563	Nm3127	210	2078784
102464	NZ19MI0083	232	2089243
71509	NZ15MI0041	224	2097979
51612	14.8703919	286	2094926
93831	Nm_3-12	293	2125605
85360	AK-2037_Str	145	1989931
71736	NZ18MI0098	187	2098753
102425	NZ19MI0036	247	2087070
102409	NZ19MI0017	266	2086298
102437	NZ19MI0048	252	2092812
71598	NZ17MI0029	203	2107008
102466	NZ19MI0085	254	2079768
92854	Nm_51-17	249	2231515
82430	20149	211	2104092
81897	20153	208	2104391
71613	NZ17MI0047	182	2129441
71799	NZNMI142	207	2124795
71757	NZNMI1327	238	2131631
71520	NZ16MI0012	205	2127341
71533	NZ16MI0027	233	2132615
71531	NZ16MI0025	207	2134516
71537	NZ16MI0034	188	2126352
71593	NZ17MI0024	226	2134750
71539	NZ16MI0036	219	2136733
71639	NZ17MI0075	218	2133890
71604	NZ17MI0036	227	2140769
71690	NZ18MI0047	198	2128352
71597	NZ17MI0028	161	2131925
71668	NZ18MI0022	182	2123451
102473	NZ19MI0092	239	2106129
102471	NZ19MI0090	233	2106390
71714	NZ18MI0074	204	2130630
102401	NZ19MI0007	236	2108330
102399	NZ19MI0004	234	2104082
93982	IE19Nm25	139	2132029
89322	M08 240537	117	2127863
61352	Nm-146	76	2109982
61351	Nm-105	116	2096473
71705	NZ18MI0064	172	2105153
71716	NZ18MI0076	220	2110611
88953	NIID669	87	2,108,927
71720	NZ18MI0080	176	2108153
71666	NZ18MI0020	173	2100928
102463	NZ19MI0082	242	2099163
71708	NZ18MI0067	173	2103067
102411	NZ19MI0019	235	2092756
71703	NZ18MI0061	169	2102093
102441	NZ19MI0053	228	2093773
71724	NZ18MI0084	208	2108471
71670	NZ18MI0024	173	2102216
71719	NZ18MI0079	178	2096664
102484	NZ19MI0105	232	2098658
102482	NZ19MI0103	236	2095033
71734	NZ18MI0096	159	2095536
102457	NZ19MI0075	231	2093723
102417	NZ19MI0025	230	2094801
102460	NZ19MI0079	234	2101673
71626	NZ17MI0062	183	2107585
71717	NZ18MI0077	184	2108405
71609	NZ17MI0041	160	2107174
102476	NZ19MI0095	253	2096387
71629	NZ17MI0065	212	2107867
102424	NZ19MI0035	226	2093802
102421	NZ19MI0029	220	2088888
102487	NZ19MI0108	230	2103977
102445	NZ19MI0057	228	2103616
102454	NZ19MI0069	236	2099152
102455	NZ19MI0071	230	2095553
71699	NZ18MI0057	177	2104257
102442	NZ19MI0054	229	2095414
102426	NZ19MI0037	235	2095429
102415	NZ19MI0023	229	2090707
71713	NZ18MI0073	207	2108136
71715	NZ18MI0075	200	2104960
71711	NZ18MI0071	198	2108650
71596	NZ17MI0027	170	2106804

FIG S1 Antibiotic susceptibility of penicillin, ciprofloxacin, and azithromycin in 87 *N. meningitidis* isolates obtained between April 1998 and March 2018. (A) Geometric mean MICs of three antibiotics determined every seven years. (B) Distribution of interpreted categories for three antibiotics based on the Clinical and Laboratory Standards Institute guideline M100-ED30.

(A)

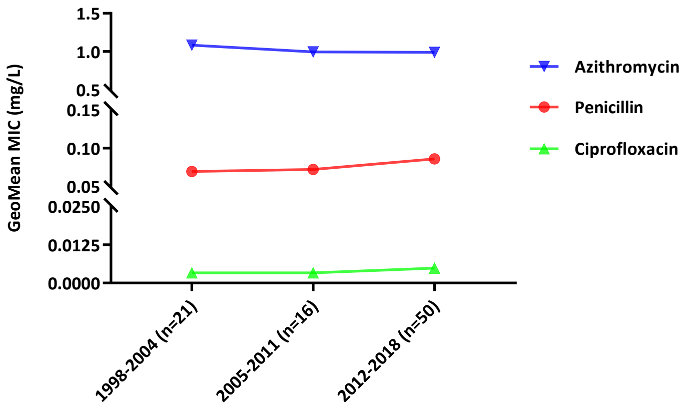

(B)

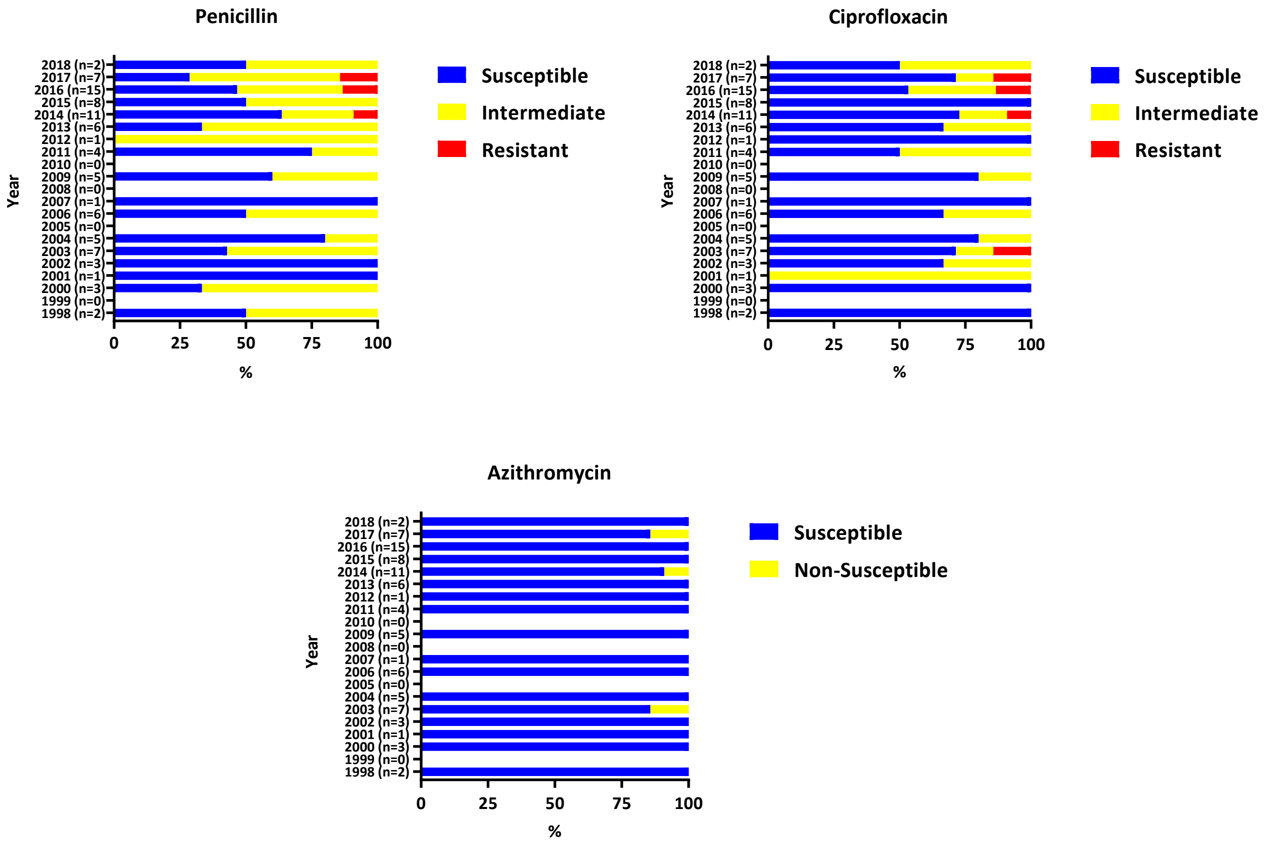

Supplement: SUPPLEMENTAL FILE 1 — Supplemental material. Download spectrum.00627-22-s001.pdf, PDF file, 0.3 MB [file spectrum.00627-22-s001.pdf]
